# Reference values for lysosomal enzymes activities using dried blood spots samples - a Brazilian experience

**DOI:** 10.1186/1746-1596-5-65

**Published:** 2010-09-29

**Authors:** Karen B Müller, Mayra DB Rodrigues, Vanessa G Pereira, Ana M Martins, Vânia D'Almeida

**Affiliations:** 1 Department of Pediatrics - Universidade Federal de São Paulo (UNIFESP), São Paulo, Brazil; 2 Department of Biosciences - Universidade Federal de São Paulo (UNIFESP), Santos, Brazil

## Abstract

**Background:**

Lysosomal storage diseases (LSD) are inherited disorders caused by deficiency of lysosomal enzymes in which early diagnosis is essential to provide timely treatment. This study reports interval values for the activity of lysosomal enzymes that are deficient in Mucopolysaccharidosis type I, Fabry, Gaucher and Pompe disease, using dried blood spots on filter paper (DBS) samples in a Brazilian population.

**Results:**

Reference activity values were obtained from healthy volunteers samples for alpha-galactosidase A (4.57 ± 1.37 umol/L/h), beta-glucosidase (3.06 ± 0.99 umol/L/h), alpha-glucosidase (ratio: 13.19 ± 4.26; % inhibition: 70.66 ± 7.60), alpha-iduronidase (3.45 ± 1.21 umol/L/h) and beta-galactosidase (14.09 ± 4.36 umol/L/h).

**Conclusion:**

Reference values of five lysosomal enzymes were determined for a Brazilian population sample. However, as our results differ from other laboratories, it highlights the importance of establishing specific reference values for each center.

## Introduction

Lysosomal storage diseases (LSD) comprise a group of more than 40 inherited disorders caused by deficiency of specific lysosomal hydrolases, which results in the accumulation of different macromolecules in the lysosome, leading to cell dysfunction and progressive clinical manifestations [1,2].

Most LSD are inherited in an autosomal recessive manner, with the exception of Hunter, Fabry and Danon diseases that are X-linked. As a group, LSD have an incidence of about 1 per 7700 live births [3].

For some LSD as Fabry, Gaucher and Pompe diseases (FD, GD and PD respectively) and Mucopolysaccharidosis type I (MPS I), enzyme replacement therapy has been widely used as a treatment option, improving the quality of life and prognosis of these patients. Since those diseases are all progressive, early diagnosis is an essential tool for a successful treatment [2].

Biochemical diagnosis of LSD is performed by determination of enzymatic activities in different biological fluids (as plasma, leukocytes, fibroblasts and most recently dried blood spots on filter paper (DBS)), using fluorimetry, immunocapture and mass spectrometry assays [4-8]. One of the advantages for the use of DBS in the diagnosis of LSD is that these samples may be transported safely through long distances, including mailing in regular envelopes, because enzyme activities remain stable for months at room temperature [8]. In addition, the preparation of a DBS sample requires only a small amount of blood [9]. These advantages are especially important in large countries like Brazil, in which there are few reference laboratories to perform these assays.

Several studies have demonstrated the feasibility of using DBS to diagnose LSD [9-11], but it is important to establish specific reference values to each center, since enzyme activity may vary due to specific characteristics of each population and mainly because different assay conditions. Therefore, the aim of this study was to establish a reference interval value for the activities of the following lysosomal enzymes in a Brazilian population sample: alpha-galactosidase A (αgal A), beta-glucosidase (βglu), acid alpha-glucosidase (αglu), alpha-iduronidase (αidua), which are used for the diagnosis of FD, GD, PD and MPS I respectively, and also beta-galactosidase (βgal), used as a quality control tool.

## Methods

This study was performed at *Laboratório de Erros Inatos do Metabolismo *(LEIM) from Universidade Federal de São Paulo (UNIFESP). Research protocols and consent forms as well as the overall investigation were ethically and scientifically approved by the Medical Research and Ethical Committee of UNIFESP (0384/05).

Brazilian healthy volunteers (HV) between the ages of 18 and 80 and without a known genetic or chronic disease were selected for our study. Samples were collected from 164 individuals (42% male and 58% female). Blood specimens were collected by venipuncture into heparin tubes and spotted on Whatman^® ^903 filter paper. Samples collected at UNIFESP were dried at room temperature for at least 4 hours and stored at the same day at 4°C in sealed plastic bags with desiccant (to prevent hydration) until analysis. Those collected at other locations were prepared and dried in the same conditions (detailed instructions were sent to those centers), sealed in plastic bags and sent to the laboratory in a maximal interval of 5 days, where they were stored at 4°C. Only high quality DBS, evaluated by their macroscopic aspect (absence of jagged edges and clots, and adequate amount of blood on the spot) and biochemically by βgal activity [9,12] were used in this work.

DBS enzyme activity assays were adapted from the methods described by Chamoles and coworkers [8,13-17]. αgal A, αglu, αidua, βgal were assayed on microplates and βglu was assayed on microtubes. For microplate assays, 1.5 mm diameter (1/16 inch) disks were punched from each DBS and placed into separate wells in a 96-well blank plate. For microtube assay, 3.0 mm diameter (1/8 inch) disks were punched from DBS and placed into separate 2.0 mL microtubes. DBS removal from the wells/tubes during the analysis was not necessary.

Reaction buffers, fluorogenic substrates, additional reagents and incubation time used for each DBS assay are described in Table 1. Assays were performed in duplicate and with one blank per sample, which was prepared by adding all reagents except the substrate. The microplate/microtube reactions and substrate solution were incubated separately at 37°C in an orbital shaker. After incubation, substrate solution was added to blanks and the stop buffer was added to all wells/tubes. The enzyme product 4-methylumbelliferone was detected (excitation 365 nm; emission 450 nm) in a fluorometer SpectraMax M2 (Molecular Devices). Fluorescence readings were corrected by blanks, and results were compared to a 4-methylumbelliferone calibration curve. Enzymatic activities were expressed as micromoles of hydrolyzed substrate per liter of blood per hour (μmol/L blood/h), with the exception of αglu, which was expressed as the ratio of neutral and lysosomal isoforms (NaG/AaGIA) and % inhibition of total acid fraction [9,17].

**Table 1:** Assay conditions for each enzyme activity assay.

ENZYME	Punch diameter	Reaction buffer	Fluorogenic substrate	Additional reagents	Incubation time	Stop buffer
alpha-galactosidase A	1.5 mm	30 μL citrate-phosphate 0.3 M pH 5.0	30 + 30* μL 4 MU-alpha-D-galactopyranoside 4.2 mM	10 μL N-acetyl-galactosamine 0.25 M	20 hours	10% ethylenediamine 1.32 M pH 11.3

alpha-iduronidase	1.5 mm	10 μL sodium formate 0.2 M pH 2.8	20 + 20* μL 4MU-alpha-L-idopyranoside 2 mM	10 μL D-saccharic acid 1,4-lactone monohydrate 3 mM	20 hours	10% ethylenediamine 1.32 M pH 11.3

beta-glucosidase	3.0 mm	30 μL citrate-phosphate 0.4 M pH 5.2	50 + 50* μL 4MU-beta-D-glycopyranoside 20 mM	40 μL sodium taurodeoxicolate hydrate 0.75%	20 hours	10% ethylenediamine 1.32 M pH 11.3

alpha-glucosidase	1.5 mm	20 μL sodium acetate 0.4 M pH 4.0 and 6.5	20 + 20* μL 4MU-alpha-D-glycopyranoside 2.8 mM	40 μL acarbose 40 μM	24 hours	10% ethylenediamine 1.32 M pH 11.3

beta-galactosidase	1.5 mm	20 μL citrate-phosphate 0.1 M pH 4.4	20 + 40* μL 4MU-beta-D-galactopyranoside 0.8 mM	20 μL sodium chloride 0.9%	3 hours	10% ethylenediamine 1.32 M pH 11.3

* addition in blank wells after incubation

MU: methylumbilliferyl

DBS samples from two healthy individuals have been used as internal controls in every reaction as well as a DBS from a confirmed patient. Within-assay coefficients of variation (CVs) were calculated from an internal control sample analyzed in all assays.

Data are expressed as mean ± standard deviation (SD). The parametric Student's *t *test was performed to compare mean activity values from both genders in HV group. The level of significance for statistical analysis was set at 0.05. All analyses were performed using the software MINITAB^® ^15.1.1.0.

## Results

βgal activity was analyzed in HV to establish its reference interval for our population and the range was 7.85 and 22.15 μmol/L blood/h (percentile 5 and 95 respectively). βgal was used as control enzyme, and DBS samples with βgal activity lower or higher than the normal range have not been included in this study, as they were considered inappropriate. The mean, standard deviation and interval values of the different enzymatic activities are described in Table 2. A significant difference between genders was observed for βglu (male: 2.82; female: 3.23; p = 0.0053) and βgal activities (male: 12.61; female: 15.17; p = 0.0001 respectively), which could be due to the higher number of female subjects in the HV group (69 males versus 95 females).

**Table 2:** Enzyme activities from HV DBS samples.

	α-gal A	α-idua	NaG/AaGIA	% inhibition	β-glu	β-gal
***n***	164	164	164	164	156	164
***mean***	4.57	3.45	13.19	70.66	3.06	14.09
***SD***	1.37	1.21	4.26	7.60	0.99	4.36
***interval***	1.44 - 10.67	1.40 - 7.78	5.03 - 28.58	36.38 - 87.08	1.51 - 7.23	4.04 - 29.65

Data expressed as μmol/L blood/h

n = number of samples

SD = standard deviation

Interval = established as minimum to maximum

% inhibition = percent of other alpha-glucosidase isoforms inhibition

Our results differ from enzymatic activity values previously described by Chamoles and coworkers, especially for αidua activity (our mean activity: 3.45; Chamoles *et al*.: 5.50) [8,13-17]. Regarding the study from Civallero and coworkers, in which several lysosomal enzymes activities were evaluated in another Brazilian sample, a difference in mean values was also observed, mainly for βglu activity (our mean value: 3.09; Civallero et al.: 4.92) [7]. This difference may be explained by sample size issues, differences in equipment, chemicals or water or even by ethnic diversity, once the Brazilian population is known to be composed of a variety of ethnic backgrounds [18]. However, these differences highlight the importance of determining specific reference values for each laboratory or reference center.

Within-assay CVs of internal controls were around 10%, interassay CVs did not exceed 20% and imprecision data are in accordance with other related studies [8,15,19,20].

## Discussion

Different biological materials have been used to diagnose LSD patients, but recently several studies have described the advantages of DBS [9,19,20]. These samples can be easily and safely transported by regular mail, even at room temperature, without altering enzyme activity [14,15]. In addition, DBS minimizes biological hazards, since the blood is impregnated in the filter paper and only a small amount of blood is required to perform the assay. Moreover, these samples do not require special storage conditions and the assay on microplate is easier to perform and less expensive than other techniques [8,9].

Although we did not evaluate newborns in this study, it is known that this group has a higher range of enzyme activity [7,8]. Therefore, it is important to emphasize that a specific range must be taken into account when analyzing newborn patients. In addition, it is important to obtain a reference interval value for this particular population if these assays would be included in newborn screening programs.

It is also extremely important to emphasize that in order to guarantee the quality of diagnosis, blood collection and DBS samples preparation must be done carefully, and the determination of a control enzyme, such as βgal or hexosaminidase should be performed as a quality control tool [9,12,14].

Although it has been demonstrated that these enzymes activities are maintained stable on DBS samples, we suggest that a sample from a healthy volunteer should be sent to the laboratory for analysis along with the samples of clinically suspected patients, which could point out any unexpected decrease of the enzymes activities during transportation [8,9]. Therefore, the establishment of reference values from healthy volunteers could be useful as an additional quality control tool for DBS assays.

We believe that the determination of enzyme activity using DBS samples is the best option for screening patients with LSD, especially in high risk groups. This alternative has several advantages that outweigh an initial evaluation of suspected subjects using DBS samples. However, as some of our results differ from other laboratories, it highlights the importance of establishing specific reference values for each reference center.

The establishment of reference values for lysosomal enzyme activities on DBS in a Brazilian population may facilitate the use of this test as a tool for LSD diagnosis in our country, and even enable the implantation of a newborn screening program.

## Competing interests

The authors declare that they have no competing interests.

## Authors' contributions

KBM, MDBR and VGP participated in the collection of samples, determination of enzyme activities, analysis and interpretation of data and drafting the manuscript. AMM helped with samples collection and the design of the study. VD participated in the design of the study, its coordination and critically revised the manuscript. All authors have read and approved the final manuscript version.
